# Improved Direction-of-Arrival Estimation of an Acoustic Source Using Support Vector Regression and Signal Correlation

**DOI:** 10.3390/s21082692

**Published:** 2021-04-11

**Authors:** Faisal Alam, Mohammed Usman, Hend I. Alkhammash, Mohd Wajid

**Affiliations:** 1 Department of Computer Engineering, Z.H.C.E.T., Aligarh Muslim University, Aligarh 202002, India; faisalalam@zhcet.ac.in; 2 Department of Electrical Engineering, King Khalid University, Abha 61411, Saudi Arabia; omfarooq@kku.edu.sa; 3 Department of Electrical Engineering, College of Engineering, Taif University, Taif 21944, Saudi Arabia; khamash.h@tu.edu.sa; 4 Department of Electronics Engineering, Z.H.C.E.T., Aligarh Muslim University, Aligarh 202002, India

**Keywords:** correlation coefficient, curve fitting, direction-of-arrival estimation, machine learning, microphone array, support vector regression

## Abstract

The direction-of-arrival (DoA) estimation of an acoustic source can be estimated with a uniform linear array using classical techniques such as generalized cross-correlation, beamforming, subspace techniques, etc. However, these methods require a search in the angular space and also have a higher angular error at the end-fire. In this paper, we propose the use of regression techniques to improve the results of DoA estimation at all angles including the end-fire. The proposed methodology employs curve-fitting on the received multi-channel microphone signals, which when applied in tandem with support vector regression (SVR) provides a better estimation of DoA as compared to the conventional techniques and other polynomial regression techniques. A multilevel regression technique is also proposed, which further improves the estimation accuracy at the end-fire. This multilevel regression technique employs the use of linear regression over the results obtained from SVR. The techniques employed here yielded an overall 63% improvement over the classical generalized cross-correlation technique.

## 1. Introduction

Applications such as hands-free mobile communication, hearing aids, target tracking, surveillance of aerial targets, etc., require a close estimation of the direction-of-arrival of a sound source [1,2,3,4,5,6,7,8,9,10,11,12,13,14,15,16,17,18]. Techniques based on microphone arrays and acoustic vector sensors (AVSs) can be employed for the accurate estimation of the DoA of the incoming acoustic wave [19,20,21,22,23,24,25,26,27]. However, the DoA estimation using the above techniques encounters practical challenges as the sound wave undergoes reflections and scattering from several objects and the surface enclosures in the surroundings. The irregular reflections of the sound waves create reverberations, and the presence of unwanted interfering sound sources causes a deterioration in the quality of the sound wave. Furthermore, ambient noise and sensor noise give rise to an additional disturbance in the acoustic wave. The estimation of the DoA can be carried out by acquiring signals impinging on a uniform linear array (ULA) of microphones/sensors. Different algorithms can be applied to the digital signals acquired on these microphones to compute the DoA. The classical known techniques/algorithms are beamforming, maximum-likelihood, the subspace method, time-difference of arrival (TDoA), etc. [28,29,30,31,32]. However, due to the presence of noise, interference and reverberations cause a higher deviation from the true value of the DoA, therefore being less suitable for many applications. In [33], the phase-mismatch error and gain-mismatch error among the sensors of the ULA were rectified using a compensated covariance matrix and phase retrieval for DOA estimation. In [34], a high-resolution, low-complexity method was presented with the use of unfolded coprime linear arrays, where the uniform property of the sub-arrays and the polynomial root finding method were used. A strategy was proposed to overcome the effect of sensor failure in a co-prime array for DoA estimation by employing the singular-value thresholding algorithm [35]. To overcome the grid-mismatch limitation, a solution was proposed in [36] that addressed the DOA estimation problem in an off-grid mode under a sparse framework. The authors in [37] reported a method for near-field and far-field localization with higher accuracy for underdetermined cases by exploiting the co-array property.

The advent of machine learning (ML) in the present era has opened up avenues for the exploration of different ML algorithms for DoA estimation [38]. In this paper, the acoustic digital signals acquired by the ULA were used to compute the feature, Pearson product moment correlation coefficients (PPMCCs). The PPMCCs paired with the known DoA were used to train the ML model for the prediction of the DoA. We carried out a comparative study for the performance of DoA estimation using multiple ML algorithms viz. linear regression, multivariate polynomial regression, and support vector regression (SVR), and compared its result with a classical technique based on the TDoA using generalized correlation coefficients (GCCs). After the assessment of the best ML model for the DoA estimation, we further proposed a curve-fitting-based pre-processing technique for improving the DoA estimation. Furthermore, a multilevel regression scheme was proposed for reducing the error in the DoA estimate at the end-fire.

The rest of the paper is organized as follows. In Section 2, the signal model is explained. Section 3 gives a brief discussion of the techniques used. In Section 4, the methodologies used are explained, and the progressive improvement with comparative results is presented in each subsection. Section 5 concludes the paper.

## 2. Signal Model

**Figure 1:**
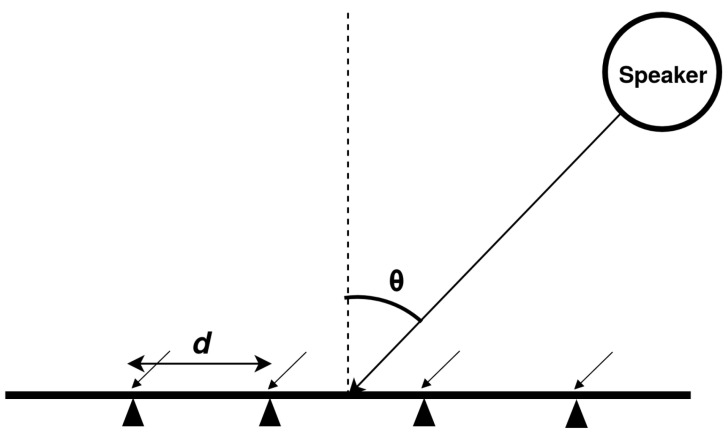
Signal impinging on a uniform linear array, where filled triangles indicate the position of omni-directional microphones.

The incoming acoustic waves moving with speed *c* were assumed to be from the source placed in the far-field; therefore, the incident wave-front was planar. The received signals were assumed to be a narrowband signal, s(t), with the center frequency *F* (where F=c/λ and λ is the wavelength). It was assumed that the sound source and the ULA were in the same plane. The DoA with respect to the normal of the ULA is denoted by θ. There were *M* number of microphones in a ULA, and each microphone was assumed to be of a point size and to have an omnidirectional pattern. The adjacent microphones in the ULA were separated by a distance *d*, as shown in Figure 1.

The signal received by the *m*th (*m* = 1 to *M*) sensor of the array was a phase shifted signal in the frequency domain, which can be formulated as:

(1) $$x_{m}\left(t\right)=s\left(t\right)e^{-j2\frac{\pi }{\lambda }D_{m}}+n_{m}\left(t\right)$$

where $D_{m}$ is the wave path difference between different microphones in the ULA, which is expressed as $D_{m}=\left(m-1\right)d\sin\theta$, and $n_{m}\left(t\right)$ is the noise added to the *m*th sensor. We can rewrite Equation (1) as:

(2) $$x\left(t\right)={\left[x_{1}\left(t\right),x_{2}\left(t\right),\ldots ..x_{m}\left(t\right)\right]}^{T}=a\left(\theta \right)s\left(t\right)+n\left(t\right).$$

where a(θ) is the steering vector of the ULA, n(t) is the noise vector, and ${\left[.\right]}^{T}$ denotes the transpose. The M×M correlation matrix $R_{\mathrm{xx}}$ of received signal vector x(t) is expressed as:

(3) $$R_{\mathrm{xx}}=E\left[x\left(t\right)x^{H}\left(t\right)\right]=a\left(\theta \right)Sa^{H}\left(\theta \right)+R_{n},$$

where E[.] and ${\left[.\right]}^{H}$ denote the ensemble average and conjugate transpose, respectively. The signal and noise correlation matrices S and $R_{n}$ can be expressed as:

(4) $$S=E\left[s\left(t\right)s^{H}\left(t\right)\right]$$

and

(5) $$R_{n}=E\left[n\left(t\right)n^{H}\left(t\right)\right],$$

respectively. We assumed that all the noise components were zero mean, mutually uncorrelated, and had the same power. Thus, we have:

(6) $$R_{n}=\sigma^{2}I,$$

where I is the identity matrix and $\sigma^{2}$ is the noise power. Then, Equation (3) can be written as:

(7) $$R_{\mathrm{xx}}=a\left(\theta \right)Sa^{H}\left(\theta \right)+\sigma^{2}I.$$

## 3. Brief Discussion of the Techniques Used

In this paper, the process of DoA estimation employed several techniques, which when applied in tandem helped the near-accurate estimation of the DoA. A brief discussion of these techniques is given in the following subsections.

### 3.1. Polynomial Regression

Linear regression is a technique that helps to find a linear relationship between predictors *x* and the response *y*. On the other hand, polynomial regression is a technique that helps identify a non-linear relationship between predictors and the response. As explained in [39], the degree of polynomial regression has to be predetermined before the training. Based on the degree of the polynomial, *n*, a parameterized equation is of the form:

(8) y=bx+ϵ,

where $b=[b_{1},b_{2}...b_{n}]$ is the parameter vector to be estimated for the best fit and $x={\left[x,x^{2},x^{3},...,x^{n}\right]}^{T}$. The non-linear terms like $x^{2}$, $x^{3}$, …, are considered to be derived dimensions based on the base dimension *x*. The polynomial regression involves performing multivariate linear regression considering higher order terms to be a separate dimension. A univariate regression is a regression involving a single predictor variable, whereas a multivariate regression involves multiple predictor variables.

### 3.2. Support Vector Regression

Support vector regression (SVR) is a technique that finds a non-linear mathematical relationship between predictors and the response where the prior information of the polynomial degree is not required [40,41]. The technique involves projecting the predictor space into a multidimensional space using a kernel function. In this work, the radial basis function (RBF) was used as the kernel function. The RBF is given as:

(9) $$K\left(x,x^{\prime }\right)=exp(-\gamma |x-x^{\prime }\left|\right),$$

where x and $x^{\prime }$ are the two vectors in the feature space. This kernel function expands into multidimensional terms. The error estimation is measured using the following loss function,

(10) $$L=\max\left\{\begin{array}{cc} 0, & |y-F(x,\hat{w})|<\epsilon \\ |y-F(x,\hat{w})|-\epsilon , & \mathrm{otherwise}. \end{array}\right.$$

As mentioned in [40], F(x,w) is a family of functions parameterized by **w**, $\hat{w}$ is that value of w that minimizes a measure of the error between **y** and F(**x**, **w**). This loss function is termed as the ϵ-insensitive loss function. It identifies a high-dimensional tube of diameter ϵ. If the estimate is within the tube, then the loss is zero; otherwise, the loss is the distance from the point of estimation to the closest tube periphery. The objective is to flatten this tube to the maximum extent possible. In the training part, linear regression was performed on this high-dimensional space. As a result of the regression process, a linear hyperplane was identified that reduced the overall loss.

The roots of the SVR method are the same as the popular support vector machine (SVM) method, which is used in classification problems and utilizes the same underlying theory. SVM cannot be directly applied here as the DoA estimation was modeled in this work as a regression problem rather than a classification problem.

### 3.3. Pearson Product Moment Correlation Coefficient

As explained in [42], the Pearson product moment correlation coefficient (PPMCC) quantifies the degree of association between two statistical variables. It also ascertains whether the variables are directly or inversely associated with each other. The PPMCC ranges between +1 and −1. The value +1 indicates the highest degree of association between the variables, which indicates that an increase in one variable is commensurate with an increase in the other variables. The PPMCC value of −1 indicates the highest degree of dissociation between the two variables where an increase in one variable is commensurate with a decrease in another variable. The values in between intervals (−1, +1) are indicative of the corresponding association between the variables proportional to the measure of their values. If *L* is the sample size, ${\left\{p_{i}\right\}}_{i=1}^{L}$ and ${\left\{q_{i}\right\}}_{i=1}^{L}$ are two variables, $\bar{p}$ is the mean of ${\left\{p_{i}\right\}}_{i=1}^{L}$, and $\bar{q}$ is the mean of ${\left\{q_{i}\right\}}_{i=1}^{L}$, then the PPMCC, $r_{pq}$, is given by:

(11) $$r_{pq}=\frac{\sum_{i=1}^{L}\left(p_{i}-\bar{p}\right)\left(q_{i}-\bar{q}\right)}{\sqrt{\sum_{i=1}^{L}{\left(p_{i}-\bar{p}\right)}^{2}}\sqrt{\sum_{i=1}^{L}{\left(q_{i}-\bar{q}\right)}^{2}}}.$$

### 3.4. Curve Fitting

Curve fitting is the process of identifying a curve that wraps around a series of data points in the best possible manner. The identification of the mathematical model or the curve starts with the proposition of a parameterized mathematical model. The objective of curve fitting identifies the value of these parameters such that the mathematical model minimizes the overall fitting error. For the best fit, the objective function has to be minimized with respect to the parameters, p, using the Levenberg–Marquardt (LM) algorithm explained in [43,44]. The LM algorithm internally uses a combination of two different methods viz. the Gradient Descent Method (GDM) and the Gauss–Newton Method (GNM). In the LM algorithm, the GDM dominates when the parameters are far apart from their actual values; however, the GNM dominates when the parameters are nearby. The goodness-of-fit is measured using the chi-squared error as given below:

(12) $$\chi^{2}\left(p\right)=\sum_{i=1}^{m}{\left[\frac{g\left(t_{i}\right)-\hat{g}\left(t_{i};p\right)}{{\sigma_{g}}_{i}}\right]}^{2}$$

where ${\sigma_{g}}_{i}$ is the average error, $\hat{g}\left(t;p\right)$ is the fitted function of independent variable *t* and a vector of *n* parameters **p**, and *m* is the number of data points in the data set.

The combination of the GDM and GNM is accomplished using a parameter λ that is tuned to fall into the appropriate method based on its magnitude,

(13) $$\left[{\frac{\partial \hat{g}}{\partial P}}^{T}\Theta \frac{\partial \hat{g}}{\partial P}+\lambda I\right]h_{m}={\frac{\partial \hat{g}}{\partial p}}^{T}\Theta \left(g-\hat{g}\right),$$

where Θ is a diagonal matrix with elements $w_{ii}=1/\sigma_{g_{i}}^{2}$ and $h_{m}$ is the perturbation parameter that reduces the chi-squared error. To reach closer to the global minimum, the first few steps are taken to be small in the steepest direction. This is accomplished by keeping the value of λ small. The equation behaves closer to the Gauss–Newton update with a small λ. If an iteration gives a high error, then λ is increased and the equation behaves like gradient descent. The scipy package in Python provides the function *curve_fit* with its optimized class that provides the curve fitting function using multiple algorithms. In this work, we used this function for the LM algorithm. Since the acoustic waves received at the microphone array were sinusoidal with additive white Gaussian noise (AWGN), to fit the curve, sinusoidal function asin(bx+ϕ) was been used where *a*, *b*, and ϕ are the parameters to be tuned to fit the curve.

## 4. Methodologies and Results

The ULA of omnidirectional microphones was used to record the spatial signals for developing the machine learning model for DOA estimation. The data set consisted of multiple recordings of microphones of a duration of 25 ms each, which were acquired for angles $0^{\circ }$, $2^{\circ }$, $4^{\circ }$, $6^{\circ }$, …, $90^{\circ }$ of a sound source with different signal-to-noise ratio (SNR) values. For each such angle with sensor noise of SNR = 26 dB, one-thousand four-hundred independent realizations of 25 ms in duration were used for training. For testing, a new data set was created with angles $0^{\circ }$ to $90^{\circ }$ with an increment of $1^{\circ }$ for SNR = 22 dB, 18 dB, 14 dB, and 10 dB. For the recorded signals at each angle, we took the PPMCC between the discrete signals from the microphones. Discrete signals from the 1st, 2nd, 3rd, and 4th microphone produced six pairs of correlations. These signals were further processed to estimate the DoA. The following subsections explain the different methodologies that were applied for DoA estimation and exhibit the result obtained with these methods. Since various techniques and methodologies were assessed for improving the DoA estimation, each succeeding subsection employs a technique that improves over the best obtained in the preceding subsection. The result is obtained in terms of root mean squared angular error (RMSAE) and $\bar{RMSAE}$ and in each subsection, and it is compared with the results in the preceding subsections. The RMSAE and $\bar{RMSAE}$ are defined in Equations (14) and (15), respectively,

(14) $$RMSAE\left(\theta \right)=\sqrt{\frac{\sum_{i=1}^{N}{\left(\theta -\theta_{i}\right)}^{2}}{N}}$$

and

(15) $$\bar{RMSAE}=\frac{1}{TOA}\sum_{\theta =0^{\circ }}^{90^{\circ }}RMSAE\left(\theta \right)$$

where $\theta_{i}$ is the *i*th prediction of the true angle θ, *N* is the total number of predictions realized, and the TOA is the total number of angles observed.

### 4.1. Regression Techniques with PPMCC

The PPMCCs were calculated between different signals $s_{1}\left(t\right)$, $s_{2}\left(t\right)$, $s_{3}\left(t\right)$, and $s_{4}\left(t\right)$ recorded by four microphones $m_{1}$, $m_{2}$, $m_{3}$, and $m_{4}$, respectively. The PPMCC between each microphone signal produced six coefficients, $c_{12}$, $c_{13}$, $c_{14}$, $c_{23}$, $c_{24}$, and $c_{34}$. These PPMCCs were used for training on the data set to produce the mathematical model of the DoA estimate. We trained the signals on the data set with SNR = 26 dB. The feature set was composed of the correlation coefficients mentioned above, and the response was the actual DoA. The regression techniques that were used and analyzed in this experiment were SVR, polynomial regression of order 1 (PR1), polynomial regression of order 2 (PR2), polynomial regression of order 3 (PR3), polynomial regression of order 4 (PR4), polynomial regression of order 5 (PR5), polynomial regression of order 6 (PR6), and polynomial regression of order 7 (PR7). In addition to these regression techniques, we also estimated the DoA for comparison with the conventional generalized cross correlation (GCC) technique.

**Figure 2:**
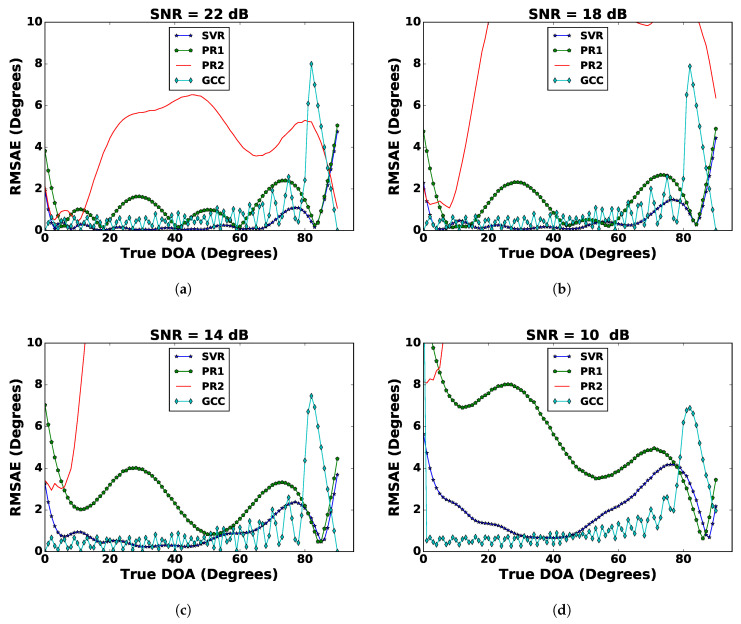
Comparison of different regression techniques (SVR, PR1, and PR2) and the GCC for (**a**) SNR = 22 dB, (**b**) SNR = 18 dB, (**c**) SNR = 14 dB, and (**d**) SNR = 10 dB.

**Figure 3:**
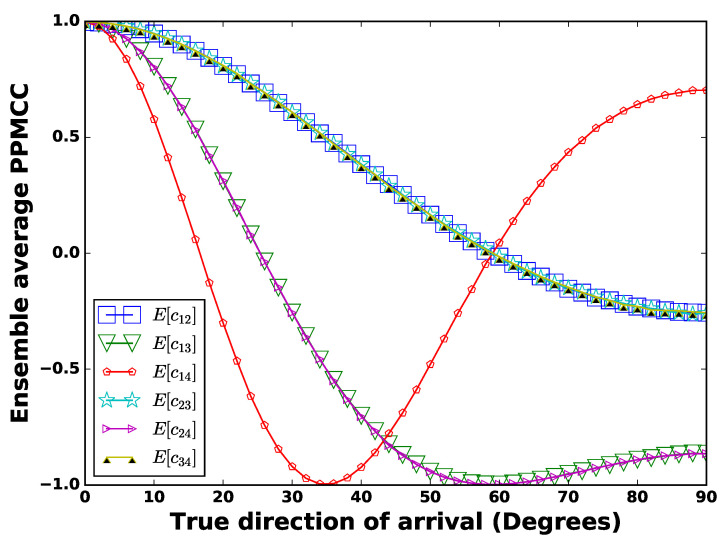
Ensemble average of the PPMCC versus the direction of arrival at SNR = 26 dB.

Figure 2 shows the comparative assessment of SVR, PR1, PR2, and GCC. Polynomial regression of orders higher than two is not shown in this figure as their RMSAE values were too high and difficult to show. In the following section, we propose a mechanism to reduce their RMSAE values. Figure 2 also reveals that the SVR gave a low RMSAE for higher SNR values, i.e., 22 dB and 18 dB. However, at lower SNR values of 14 dB and 10 dB, the GCC was robust. Moreover, the GCC provided an RMSAE closer to SVR even on higher SNR values. In short, the GCC was a better estimator as it consistently gave good approximation at low, as well as high SNR values (the next section improves the performance of ML algorithms). Among the regression techniques, SVR performed better than PR1, which in turn performed better than PR2. The $\bar{RMSAE}$ of all the regression techniques and GCC are shown in Table 1. Another observation was that the regression techniques had a higher RMSAE at the broadside (ranging between $0^{\circ }$ to $5^{\circ }$), as well as at the end-fire (ranging from $80^{\circ }$ to $90^{\circ }$). The cause for higher $\bar{RMSAE}$ near the end-fire was identified as follows. Consider the data set with SNR = 26 dB: the PPMCCs were computed. Then, we took the ensemble average of PPMCCs ($E[c_{12}]$, $E[c_{13}]$, $E[c_{14}]$, $E[c_{23}]$, $E[c_{24}]$, and $E[c_{34}]$) for each DoA, which is shown in Figure 3. This revealed that the correlations at the end-fire were steady with almost the same values, which was due to a smaller change in the relative time-delay with respect to the DoA. The relative time-delay, $\tau_{i,i+1}$, between the signals of two adjacent microphones ($m_{i}\left(t\right)$ and $m_{i+1}\left(t\right)$) for a planar wave-front are approximated by:

(16) $$\tau_{i,i+1}\approx \frac{d}{c}sin\left(\theta \right),$$

where *d* is the microphone separation, *c* is the speed of sound, and θ is the direction of arrival of a planar wave with respect to the axis normal to the ULA. The rate of change of relative delay with respect to θ is given by:

(17) $$\frac{d\tau_{i,i+1}}{d\theta }\approx \frac{d}{c}cos\left(\theta \right),$$

which shows that at $\theta \approx 90^{o}$, the $\frac{d\tau }{d\theta }$ is small, therefore the rate of change of the PPMCC is also small. This steady value of $\tau_{i,i+1}$ caused the same values of all ensemble PPMCC over a span of DoAs, thereby causing the regression techniques to have an error in the estimation of the DoA near end-fire. For the no noise case, the signal at all microphones would have similar waveforms, which would indicate that the PPMCC should be unity; however, random noise at the microphones caused the random variation of the PPMCC, and thereby, the learning/training of the machine was poor from the data at the broadside, hence the higher $\bar{RMSAE}$.

### 4.2. Improvement with Curve Fitting

**Figure 4:**
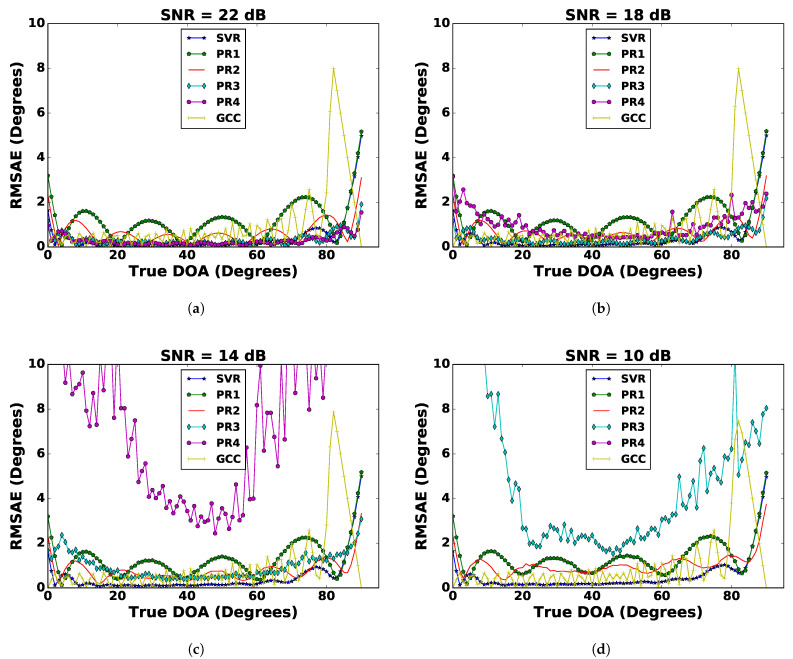
Comparison of different regression techniques (SVR, PR1, and PR2) and the GCC with curve fitting for (**a**) SNR = 22 dB, (**b**) SNR = 18 dB, (**c**) SNR = 14 dB, and (**d**) SNR = 10 dB.

**Figure 5:**
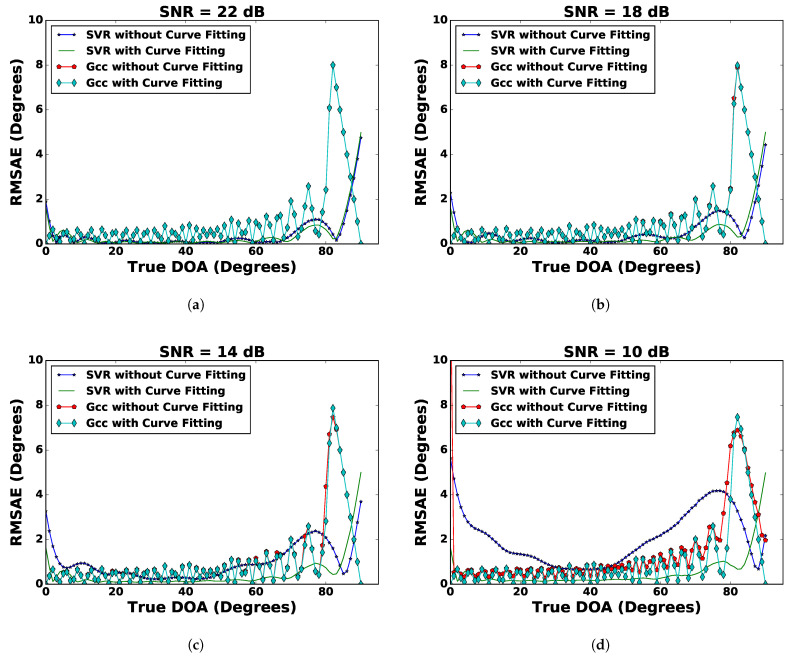
Comparison of SVR and the GCC with and without curve fitting for (**a**) SNR = 22 dB, (**b**) SNR = 18 dB, (**c**) SNR = 14 dB, and (**d**) SNR = 10 dB.

To improve on the DoA estimation using regression techniques, curve fitting was applied on the noisy sinusoidal signal recorded by the microphones, which was described in Section 3.4. The process of curve fitting reduced the noise in the recorded signals, thereby providing a sanitized input for improved results. After pre-processing with curve fitting on the recorded noisy signals, the regression techniques mentioned in Section 4 were applied. The RMSAE versus DoA results with the SNR ranging from 22 to 10 dB with a decrement of 4 dB are shown in Figure 4. It also shows the results obtained from the GCC for comparison. A closer look at the results reveals that pre-processing with curve fitting reduced the RMSAE of the DoA estimates with regression techniques. Amongst all the regression techniques, SVR outperformed then in terms of the RMSAE. Polynomial regression of higher order yielded poor results of DoA estimation without curve fitting with a too high RMSAE. It can be seen that after pre-processing with curve fitting, the RMSAE of the DoA estimate was significantly reduced. The GCC continued to perform better with a consistently lower RMSAE, but it had a highly rugged curve with respect to the true DoA. Figure 5 shows the performance comparison of SVR and the GCC with and without curve fitting. It was observed that SVR with curve fitting had a lower RMSAE. A comparison in terms of $\bar{RMSAE}$ of different regression techniques and the GCC with and without curve fitting is shown in Table 1. It can be seen from this table that the $\bar{RMSAE}$ of SVR with curve fitting had the lowest values for each experiment with particular SNR values of 22 dB, 18 dB, 14 dB, and 10 dB. In contrast to the GCC, as the SNR value decreased, the curve fitting showed more improvement in the performance of SVR.

### 4.3. DoA Estimation Improvement at the End-Fire

As mentioned in Section 4.1, it can be observed from all the preceding results that the RMSAE error was consistently high at the end-fire ranging from $80^{\circ }$ and above. To mitigate this high error, the bias and standard deviation analysis of the DoA estimates were performed. Based on this analysis, we proposed a multilevel regression to reduce the bias and further improve the accuracy at the end-fire.

#### 4.3.1. Analysis of the DoA Estimation: Bias and Standard Deviation

**Figure 6:**
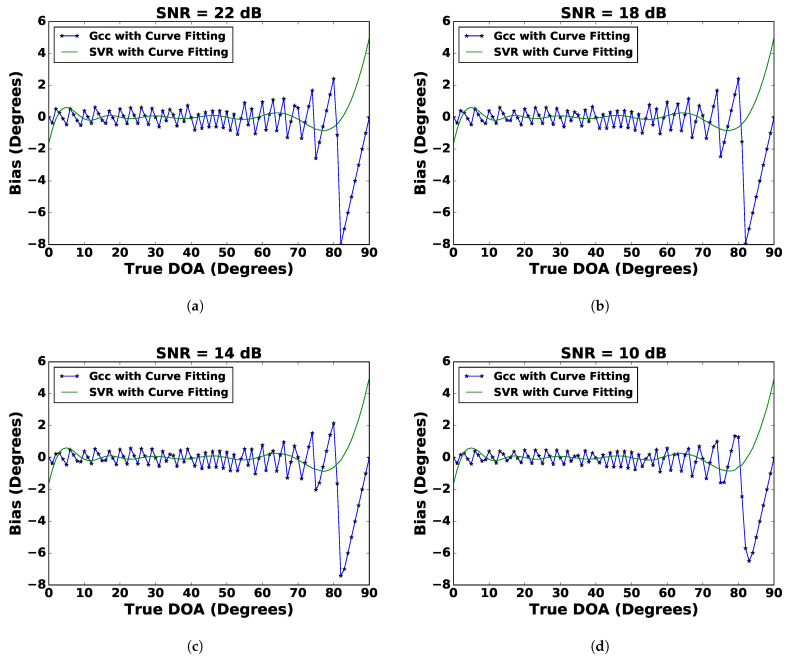
Bias comparison of SVR with curve fitting and the GCC with curve fitting for (**a**) SNR = 22 dB, (**b**) SNR = 18 dB, (**c**) SNR = 14 dB, and (**d**) SNR = 10 dB.

**Figure 7:**
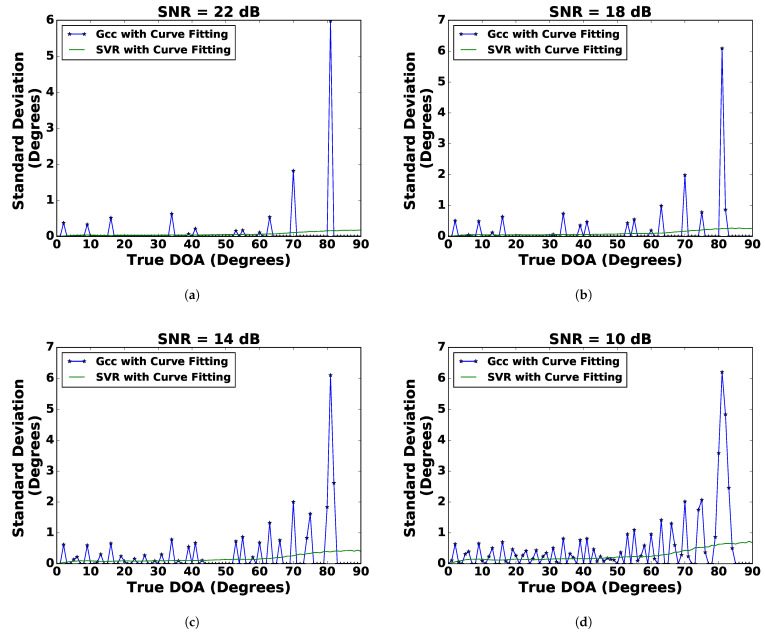
Comparison of the standard deviation of SVR with curve fitting (SVR-CF) and the GCC with curve fitting for (**a**) SNR = 22 dB, (**b**) SNR = 18 dB, (**c**) SNR = 14 dB, and (**d**) SNR = 10 dB.

The bias of a DoA estimator is the difference between the estimated DoA and the true DoA. If the bias of a DoA estimator is a certain value, then the estimation can be improved by subtracting the bias from the estimated value. The standard deviation of the DoA estimate indicates how much an estimated DoA differs from its expected value, which is a random error and cannot be compensated for a given estimate. Figure 6 and Figure 7 show the bias and standard deviation of the DoA estimation for SVR and the GCC with curve fitting. It can be observed from Figure 6 and Figure 7 that the bias for SVR was stable, whereas the bias for the GCC fluctuated with respect to true DoA. This stable bias in the SVR was beneficial as it aided in bias error compensation. The standard deviation of the DoA estimate for SVR was relatively more stable than the GCC. In contrast to SVR, the standard deviation of the DoA estimate for the GCC showed spikes. Therefore, it can be concluded that SVR with curve fitting provided the best DoA estimate amongst all techniques considered.

#### 4.3.2. Improved DoA Estimation with Multilevel Regression

**Figure 8:**
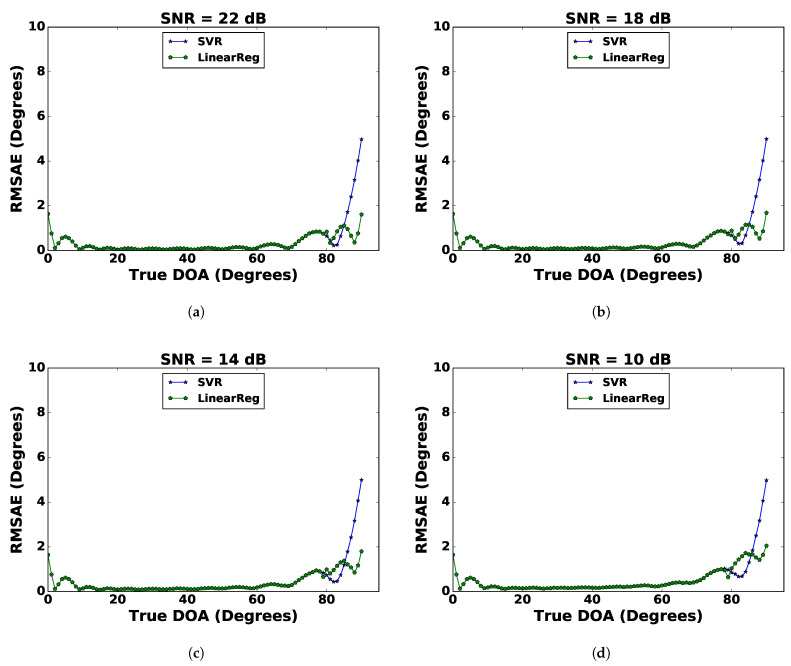
Comparison between SVR-CF and SVR-CF-LR for the DoA estimate in terms of the RMSAE at the end-fire for (**a**) SNR = 22 dB, (**b**) SNR = 18 dB, (**c**) SNR = 14 dB, and (**d**) SNR = 10 dB.

It can be observed from Figure 4 that despite curve fitting, the DoA estimation error in the range between $80^{\circ }$ and $90^{\circ }$ (end-fire) was relatively higher as compared to the DoA ranging between $0^{\circ }$ and $80^{\circ }$. Even after applying curve fitting, there was no substantial improvement in DoA estimation at the end-fire. Figure 6 reveals that the SVR bias curve for DoA estimation was increasing near the end-fire. To remove this bias, a second-level regression model (SLRM) was applied at the end-fire. The input to the SLRM was the DoA estimated from the SVR with curve fitting (SVR-CF) model, and the output was expected to compensate for this bias. The SLRM was trained with linear regression (LR) for angles estimated to be between $80^{\circ }$ and $90^{\circ }$ at SNR = 26 dB. The response from this second-level regression for (a) SNR = 22 dB, (b) SNR = 18 dB, (c) SNR = 14 dB, and (d) SNR = 10 dB is shown in Figure 8. It was observed that the RMSAE was reduced significantly at the end-fire with the maximum RMSAE reducing from $6^{\circ }$ to $2^{\circ }$ by this improvement. Table 2 compares the results of the SVR-CF model and the SVR-CF tandem with LR (SVR-CF-LR) model in terms of $\bar{RMSAE}$. It can be seen that the $\bar{RMSAE}$ values significantly reduced, indicating the SLRM further improved the DoA estimation.

## 5. Conclusions

DoA estimation of a sound source has many applications such as unmanned aerial vehicles, hearing aids, surveillance, etc. Many techniques such as beamforming and the GCC, employing a uniform linear array of microphones, have been studied in the past. However, these techniques have not been as effective and produced errors in the estimated angle owing to the impact of noise that blends with the received signals. This work aimed at increasing the efficacy of DoA estimation by exploring multiple machine learning techniques. The models for polynomial regression of order one to order seven and SVR were trained with the PPMCC as the feature selected, then compared with the classical GCC technique for comparative analysis. Since the results were not very impressive, we explored pre-processing techniques on the incoming signals for improved results. Curve-fitting as the pre-processing technique was applied, and the same regression models PR 1-7 and SVR were trained and tested after pre-processing and compared with each other. Among the regression techniques used, SVR fared the best with a minimum error when compared with the PR techniques and the GCC. However, all the techniques were shown to yield high errors near the end-fire. To reduce the error at the end-fire, multilevel regression was applied with linear regression as the second-level regression on the results of SVR. This technique proved to be nearly accurate, stable, and unbiased and produced approximately a 63% improvement in the estimated angle when compared with the classical GCC technique.

## Figures and Tables

**Table 1:** $\bar{RMSAE}$ (degrees) values with multiple regressions and conventional techniques.

Base Technique	SNR (dB)	$\bar{\mathrm{RMSAE}}$ (Degrees)	
		Without Curve Fitting	With Curve Fitting
SVR	22	0.428	0.41
	18	0.551	0.429
	14	0.966	0.464
	10	2.039	0.53
PR1	22	1.212	1.194
	18	1.418	1.217
	14	2.587	1.264
	10	5.567	1.363
PR2	22	4.23	0.655
	18	10.351	0.687
	14	25.232	0.772
	10	59.379	1.034
PR3	22	22.086	0.326
	18	Too High	0.398
	14	Too High	0.948
	10	Too High	4.811
PR4	22	Too High	0.286
	18	Too High	0.983
	14	Too High	7.95
	10	Too High	73.604
PR5	22	Too High	0.27
	18	Too High	1.932
	14	Too High	19.671
	10	Too High	High
PR6	22	Too High	0.275
	18	Too High	2.183
	14	Too High	21.895
	10	Too High	High
PR7	22	Too High	0.326
	18	Too High	2.967
	14	Too High	31.947
	10	Too High	High
GCC	22	0.983	0.983
	18	0.988	0.988
	14	0.998	0.998
	10	1.027	1.027

**Table 2:** $\bar{RMSAE}$ of the DoA estimate for SVR-CF and SVR-CF-LR at the end-fire.

Technique	SNR (dB)	$\bar{\mathrm{RMSAE}}$ (Degrees)
SVR-CF	22	0.410
	18	0.429
	14	0.464
	10	0.530
SVR-CF-LR	22	0.294
	18	0.322
	14	0.375
	10	0.473

## Data Availability

Data sharing not applicable.

## Abbreviations

The following abbreviations are used in this manuscript:

DoA	Direction of arrival
SVR	Support vector regression
AVS	Acoustic vector sensor
ULA	Uniform linear array
TDoA	Time-difference of arrival
ML	Machine learning
PPMCC	Pearson product moment correlation coefficient
GCC	Generalized correlation coefficient
SVM	Scalable vector machine
LM	Levenberg–Marquardt
RBF	Radial basis function
GDM	Gradient descent method
GNM	Gauss–Newton method
SNR	Signal-to-noise ratio
AWGN	Additive white Gaussian noise
PRn	Polynomial regression of order n
SLRM	Second-level regression model
SVR-CF	SVR with curve fitting
SVR-CF-LR	SVR-CF in tandem with linear regression
